# Integrated community case management: planning for sustainability in five African countries

**DOI:** 10.7189/jogh.09.010802

**Published:** 2019-06

**Authors:** Jennifer Yourkavitch, Lwendo Moonzwe Davis, Reeti Hobson, Sharon Arscott-Mills, Daniel Anson, Gunther Baugh, Salim Sadruddin, Jean-Caurent Mantshumba, Bacary Sambou, Jean Tony Bakukulu, Pascal Ngoy Leya, Misheck Luhanga, Leslie Mgalula, Gomezgani Jenda, Humphreys Nsona, Santos Alfredo Nassivila, Eva de Carvalho, Marla Smith, Moumouni Absi, Fatima Aboubakar, Aminata Tinni Konate, Mariam Wahab, Joy Ufere, Chinwoke Isiguzo, Lynda Ozor, Patrick B Gimba, Ibrahim Ndaliman

**Affiliations:** 1 ICF, Rockville, Maryland, USA; 2 Independent Consultant, Silver Spring, Maryland, USA; formerly ICF, Rockville, Maryland, USA; 3 World Health Organization, Geneva, Switzerland; 4 Independent consultant for ICF, Kinshasa, Democratic Republic of Congo; 5 World Health Organization, Kinshasa, Democratic Republic of Congo; 6 IMNMCI Program, Kinshasa, Democratic Republic of Congo; 7 Abt Associates; formerly International Rescue Committee, Kinshasa, Democratic Republic of Congo; 8 Independent consultant for ICF, Lilongwe, Malawi; 9 World Health Organization, Lilongwe, Malawi; 10 Save the Children, Lilongwe, Malawi; 11 Ministry of Health, Malawi; 12 Independent consultant for ICF, Maputo, Mozambique; 13 World Health Organization, Maputo, Mozambique; 14 Save the Children, Maputo, Mozambique; 15 Independent consultant for ICF, Niamey, Niger; 16 World Health Organization, Niamey, Niger; 17 Ministère de la Santé Publique, Niamey, Niger; 18 Independent consultant for ICF, Abuja, Nigeria; 19 World Health Organization, Abuja, Nigeria; 20 Society for Family Health, Abia State, Nigeria; 21 State Ministry of Health, Niger State, Nigeria; 22 Malaria Consortium, Niger State, Nigeria

## Abstract

**Background:**

The World Health Organization (WHO) launched an initiative to plan for the sustainability of integrated community case management (iCCM) programmes supported by the Rapid Access Expansion (RAcE) Programme in five African countries in 2016. WHO contracted experts to facilitate sustainability planning among Ministries of Health, WHO, nongovernmental organisation grantees, and other stakeholders.

**Methods:**

We designed an iterative and unique process for each RAcE project area which involved creating a sustainability framework to guide planning; convening meetings to identify and prioritise elements of the framework; forming technical working groups to build country ownership; and, ultimately, creating roadmaps to guide efforts to fully transfer ownership of the iCCM programmes to host countries. For this analysis, we compared priorities identified in roadmaps across RAcE project sites, examined progress against roadmaps via transition plans, and produced recommendations for short-term actions based on roadmap priorities that were unaddressed or needed further attention.

**Results:**

This article describes the sustainability planning process, roadmap priorities, progress against roadmaps, and recommendations made for each project area. We found a few patterns among the prioritised roadmap elements. Overall, every project area identified priorities related to policy and coordination of external stakeholders including funders; supply chain management; service delivery and referral system; and communication and social mobilisation, indicating that these factors have persisted despite iCCM programme maturity, and are also of concern to new programmes. We also found that a facilitated process to identify and document programme priorities in roadmaps, along with deliberately planning for transition from an external implementer to a national system could support the sustainability of iCCM programmes by facilitating teams of stakeholders to accomplish explicit tasks related to transitioning the programme.

**Conclusions:**

Certain common elements are of concern for sustaining iCCM programmes across countries, among them political leadership, supply chain management, data processes, human resources, and community engagement. Adapting and using a sustainability planning approach created an inclusive and comprehensive dialogue about systemic factors that influence the sustainability of iCCM services and facilitated changes to health systems in each country.

Sustainability, or “the extent to which an evidence-based intervention is able to deliver its intended benefits after external support from a donor agency is terminated” [1], should be the end goal of most donor-funded global health interventions. Sustainability planning aims to facilitate the transition and may employ a specific transition plan for that purpose, leading to the formal handover of a donor-funded programme to a local partner. Although sustainability planning has been promoted in global health programmes, and, more recently, incorporated into strategies to strengthen countries’ self-reliance [2], it has historically received limited resources because funding is focused on programme planning, implementation, monitoring, and evaluation. Nonetheless, the sustainability of positive health outcomes continues to gain importance in the current global context, with reduced funding for development programmes and increasing recognition of the need for processes to transition them to country ownership [3-5]. In addition, a drive towards universal health coverage means that capitalizing on integrated health services and decreasing redundant or parallel efforts in health programming is paramount.

Integrated community case management (iCCM) of childhood pneumonia, diarrhoea, and malaria has increased access to treatment for children under five years of age, and notably reduced mortality in areas of limited health services [6]. A hallmark feature of iCCM programmes is the use of trained community health workers (CHWs) that can deliver diagnostic and treatment services for multiple childhood illnesses [7,8]. As a health care service delivery strategy, iCCM includes the training, supplying, and supervising of CHWs to treat children for diarrhoea using oral rehydration salts (ORS), to treat children for suspected pneumonia using oral antibiotics, and to administer rapid diagnostic tests and treat children for malaria using artemisinin-based combination therapy [9].

Given the focus of the Sustainable Development Goals (SDG), particularly SDG 3.2 (ending preventable deaths of newborns and children under 5 years of age) and SDG 3.8 (achieving universal health coverage), more countries are scaling-up iCCM to strategically increase access to essential health services. Understanding how iCCM has been implemented is therefore imperative to sustain and scale achievements in iCCM service delivery [10,11]. Questions around feasibility of sustainability in the long-term persist, particularly in light of donor-funded programmes [11-16]. Except for a few countries, iCCM programmes have been mainly funded by donors, putting the sustainability of such programmes at risk due to reliance on external funding.

Substantial research on the critical elements required for sustainable health programmes already exist, in particular, for HIV/AIDS programmes. The US President’s Emergency Plan for AIDS Relief (known as PEPFAR) 3.0 presided over a shift in HIV/AIDS programming to a more sustainable and country-owned approach, with a focus on countries and key populations with high disease burden [17]. This focus on transition has provided examples of implementation of key elements required for sustainability, some of which are generalisable to other health areas, including: leadership and management capacity, political and economic factors, supportive policies, alternative funding sources, integration of programmes into the wider health system, institutionalization of processes, the strength of procurement and supply chain management, and identification of staffing and training needs, amongst others [17]. Analyses have indicated that current spending on AIDS is not sufficient to sustain achievements, necessitating a strategic approach to programme and sustainability planning, so that low-income countries can reliably manage HIV programming [18].

Lessons from transitioning large-scale HIV/AIDS programmes parallel those derived from iCCM programme research. George et al. [15] emphasises the importance of iCCM policy analyses to identify and understand factors that pose challenges to achieving and sustaining scale, and others advocate for including local perspectives and evidence [19,20]. Government support and political will, stable funding of financial support, organisational and contextual factors, community support, commodities and supplies, and human resources including management capacity are also identified as critical elements of sustaining the health gains made through iCCM [12-14,21-23]. Like sustained health behavior change, programme sustainability is multidimensional, with both internal and external factors affecting it [24].

WHO’s Rapid Access Expansion (RAcE) Programme increased access to treatment for malaria, pneumonia, and diarrhoeal disease among children under five years of age through iCCM in five countries: the Democratic Republic of the Congo (DRC), Malawi, Mozambique, Niger, and Nigeria, as part of WHO’s Global Malaria Programme, with funding from Global Affairs Canada (**Table 1**). The RAcE programme also aimed to strengthen the capacity of national and local health authorities to manage and implement iCCM activities in all five countries. WHO recognized that a systematic and inclusive process to plan for the sustainability and transition of iCCM interventions was crucial to sustain the achievements in reducing child mortality in each of the RAcE project areas. WHO contracted experts in 2016 to facilitate a planning process by providing technical assistance to national, state, and local health authorities, communities, and other key stakeholders to develop a sustainability strategy. The main documents produced included a roadmap consistent with national priorities and the many guidelines for the health system, particularly child and community health, and a plan to transition management and service delivery of RAcE activities to national structures. The purpose of this article is to describe the sustainability planning process for the iCCM programmes and its outputs, and to examine progress against the roadmap to transition iCCM programmes in RAcE countries.

**Table 1:** RAcE programme grantees, local partners, region of implementation, and child health context

Local partner	Implementation region	Child health context	iCCM context
Democratic Republic of Congo, International Rescue Committee			
**Ministère de la Santé Publique**	11 health zones of Tanganyika Province	104 deaths per 1000 live births [25]	Introduced in 2003, but uneven progress [26]; RAcE brought renewed emphasis in 2013.
		Leading causes of death for children under five: diarrhoea (11%), malaria (15%) and pneumonia (16%) [27]	CHWs (called *Relais Communautaire*) are supported by a primary health care strategy and a three-level pyramid system—a central level (top of pyramid), an intermediate level (provinces and districts), and a peripheral level (health zones), which oversee health services. A national Ministry of Health (MOH)-led iCCM Task Force established during the RAcE programme provides overall guidance
		Treatment was sought for only about half of children under five who had fever in 2014; 6% of children with fever received artemisinin combination therapy; less than half of children under five who had diarrhea in the two weeks preceding the survey received oral rehydration therapy [25]	
Malawi, Save the Children			
**Ministry of Health**	Eight districts: Dedza, Likoma, Lilongwe, Mzimba North, Nkhata Bay, Ntcheu, Ntchisi, and Rumphi	63 deaths per 1000 live births [28]	Began in 2009, building on IMCI programme.
		Leading causes of death in children under five in 2015 included pneumonia, diarrhoeal diseases and malaria [29]	Focuses on hard-to-reach areas more than eight kilometres from a health facility
		In 2015/16, caregivers of 67% of children under five with fever sought advice or treatment, and 35% of those children received artemisinin combination therapy. Caregivers of 60% of children under five with diarrhoea sought treatment from a health facility, and 65% of those children received ORS [28]	CHWs (called Health Surveillance Assistants) are recruited and salaried by MOH [30]
			The MOH IMCI unit, in collaboration with the Community-based Primary Health Care Programme and district teams, is responsible for oversight and implementation.
Mozambique, Save the Children			
**Malaria Consortium and Ministério da Saúde**	Four provinces: Inhambane, Manica, Nampula, and Zambezia	82 deaths per 1000 live births [31]	Since 1978, the MOH (MISAU) has trained CHWs (*Agentes Polivalentes Elementares de Saúde* (APEs)) to increase access to health care
		Leading causes of child death: malaria (13%), pneumonia (14%), and diarrhoea (9%) [31]	By the end of 2013, MISAU and its implementing partners had trained more than 2200 APEs in iCCM [32]
		In 2011, caregivers of 56% of children under five with a fever sought treatment, and 18% of those children received artemisinin combination therapy.[33] Of the 56% of children under five for whom advice or treatment for diarrhoea was sought, 55% received ORS [33]	MISAU oversees APEs who provide preventative, curative, and referral services to communities across the country.
Niger, World Vision			
**Ministère de la Santé Publique**	Dosso region: Boboye, Dosso, and Doutchi districts; Tahoua region: Keita district	104 deaths per 1000 live births [34]	iCCM was adopted in 2005 using *Agents de Sante Communautaire*, but implementation was limited
		Main causes of death for children under five in 2015 included malaria (11%), pneumonia (21%), and diarrhoea (11%) [35]	Through the RAcE programme, more than 1200 CHWs called *Relais Communautaires* (RComs) have been trained to diagnose and treat or refer malaria, pneumonia, and diarrhoea cases among children under five
		In 2012, caregivers of 64% of children with fever sought advice or treatment, but only 15% of those children received artemisinin combination therapy. 51% of children under five with diarrhoea were taken to a health facility, and 44% of those children received ORS [36]	The government oversees the iCCM programme
Abia State, Nigeria: Society for Family Health; Niger State, Nigeria: Malaria Consortium			
**State Ministry of Health and the Abia State Primary Health Care Development Agency**	Fifteen of 17 local government areas	128 deaths per 1000 live births in Nigeria [37]	iCCM was introduced by RAcE in 2012
		58% of child deaths in Nigeria caused by malaria, pneumonia, and diarrhea [37]	CHWs (community-oriented resource persons (CORPs)) provide case management in communities
		Caregivers in Abia and Niger States sought treatment for about one-third of fever cases for children under five [37]	The Federal Ministry of Health established the National iCCM Task Force and subcommittees and developed national guidelines on iCCM
**State Ministry of Health and the Niger State Primary Health Care Development Agency**	Six local government areas		

ORS – oral rehydration salts, iCCM – integrated community case management, CHW – community health worker, MISAU - Ministerio da Saude, APE – Agentes Polivalentes Elementares de Saúde, RComm – *Relais Communautaires*

## METHODS

This article describes a programme planning process and examines progress made against that plan; it is not research involving human subjects, so we did not seek ethics approval. We approached the challenge of sustaining iCCM programmes by incorporating recommended themes of adaptation and a learning health care system [38] in an established dynamic sustainability planning process [39]. We utilised a sustainability framework focused on six components, to facilitate a series of defined steps to coordinate the local system of people and institutions managing, providing, and influencing iCCM services in each RAcE programme area. This awareness of health system dynamics and interactions within a local system, where people and institutions naturally and strategically adapt to one another and change in capacity in a nonlinear fashion [40], underscores the importance of coordinating and collaborating with both national and local stakeholders.

### Creating a sustainability framework for iCCM services

**Figure 1:**
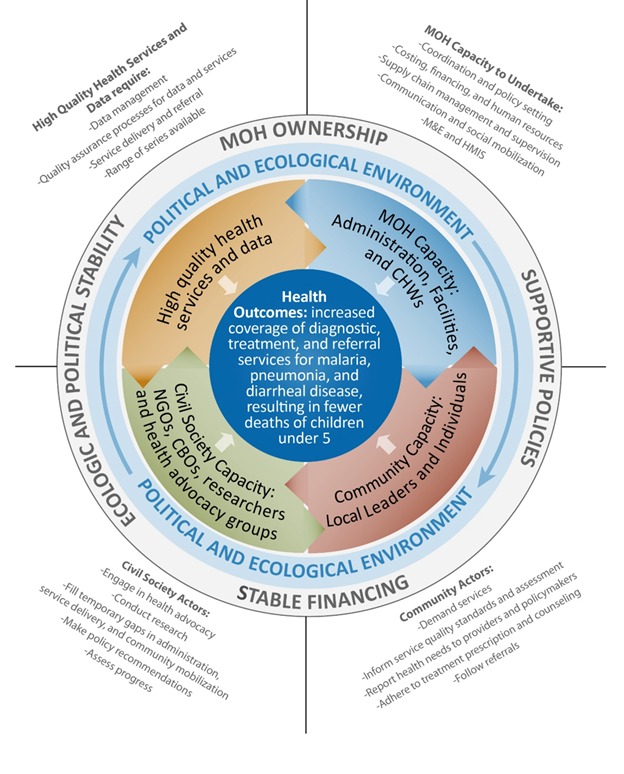
Sustainability framework for integrated community case management (iCCM).

We drafted a sustainability framework to guide the planning, identifying themes and components incorporated in previous work with sustainability planning for maternal and child health programmes [39] and literature pertinent to iCCM programmes [41]. The framework draft depicted six components, each comprising several elements. We presented the draft framework for discussion and validation to key RAcE project stakeholders (MOH, WHO, and NGO grantee staff) from the DRC, Malawi, Mozambique, Niger, and Abia and Niger States, Nigeria at the June 2016 inception meeting held in Abuja, Nigeria and subsequently refined the framework with feedback obtained at that meeting. We validated this framework with a larger group of stakeholders at workshops in each RAcE project area to create the final guiding framework for this sustainability planning initiative (**Figure 1** and Appendix S1 in **Online Supplementary Document**).

### Sustainability workshops

We organized and facilitated sustainability planning workshops in each country with support from NGO grantees and WHO country offices. The three-day workshops had two objectives: to draft a roadmap for institutionalising iCCM, and to draft a transition plan in support of the roadmap to guide activities during the last year of RAcE support. During the workshops, key stakeholders, including MOH, WHO, and NGO grantee staff, along with other influential government, academic, donor, and civil society actors, created a statement describing the national vision for the iCCM programme (Appendix S2 in **Online Supplementary Document**). To create a vision statement, facilitators prompted participants to form small groups and create a narrative or picture of what a sustained iCCM programme would look like two years after the end of the RAcE project, taking on the role of a post-project evaluation team conducting an evaluation on how well aspects of the iCCM programme had been sustained. Small groups then presented their visions to the full group. Facilitators and participants mapped commonalities and discussed differences to reach consensus on the vision statement.

At the workshop, participants also validated the sustainability framework and included points to contextualize it to their particular setting in the roadmap and transition plans (sample workshop agenda in Appendix S3 in **Online Supplementary Document**). For this purpose, roadmaps were conceptualized as documenting both the current state of sustainability of the iCCM programmes and the milestones or benchmarks that, if achieved, would enhance sustainability. We created a roadmap template to guide participants’ discussions and work. Participants worked in small groups to identify issues, next steps, and timelines related to one or more components of the sustainability framework. These groups also drafted detailed transition plans to guide activities during the next year. Transition plans typically aligned with the first year of activities articulated in the roadmaps. In each programme area, state or national authorities approved the roadmaps.

### Monitoring progress towards sustainability

We monitored the status of activities through progress update meetings with NGO grantees and related reports for three months for all project areas except Mozambique, where the project ended after the roadmap was completed, and then analyzed progress by synthesizing information from key informants and monitoring reports. We produced a synthesis report for each RAcE project area and co-facilitated dissemination meetings for participants to discuss the findings presented in the reports and to update the roadmaps.

For this analysis, we compared priorities identified in roadmaps across RAcE project sites and examined progress against roadmaps during the monitoring period and recommendations based on roadmap priorities that were unaddressed or needed further attention.

## RESULTS

This section presents the main outputs of the processes undertaken: the sustainability framework for iCCM, vision statements, and roadmap priorities; and, synthesizes information about progress made against the roadmaps and future priorities for all RAcE sites. We present the planning process for Niger State, Nigeria as a detailed case study in Appendix S4 in **Online Supplementary Document**.

### Sustainability framework for iCCM

The validated sustainability framework for iCCM comprises six components: health outcomes, high-quality health services and data, MOH capacity, civil society capacity, community capacity, and the political and ecological environment. Each component is supported by several elements (**Figure 1**). Health outcomes, at the center, are affected by interactions among high-quality health services and data, MOH capacity, community capacity and civil society capacity, which operate within a political and ecological environment that directly affects the iCCM programme and its sustainability but is only indirectly affected by it. The same framework guided planning in all of the project areas except Malawi, where the team proposed minor adaptations (Appendix S1 in **Online Supplementary Document**). Stakeholders in Malawi wanted to encircle the health outcomes with high-quality health services, supported by MOH capacity, civil society capacity, and community capacity. In addition, they included culture and communication in the political and ecological environment. The components and elements in the sustainability framework guided the planning reflected in the roadmaps.

### Comparative analysis of roadmap priorities

We compared roadmap priorities (**Table 2**) and identified a few patterns among the prioritised elements in different project areas. Overall, every project area identified priorities related to policy and coordination of external stakeholders including funders; supply chain management; service delivery and referral system; and communication and social mobilisation, indicating that these factors have persisted despite programme maturity, and are also of concern to new programmes. Four programme areas identified internal planning and coordination; supervision; and, quality assurance for services as priorities. Countries with either a mature iCCM programme (Malawi) or mature CHW programme (Mozambique) did not identify general human resource issues, including training, capacity building and recruitment, as a priority, which emerged as a priority for the other programme locations. However, stakeholders in Malawi identified specific human resource issues pertaining to CHW deployment, and internal planning and coordination remains a challenge in Mozambique. Data management, including data use, was identified as a priority for the newer iCCM programmes, including the programmes in Niger, and Abia and Niger States in Nigeria.

**Table 2:** Prioritised roadmap elements and locations

	DRC	Malawi	Mozambique	Niger	Abia State, Nigeria	Niger State, Nigeria
Financing	X		X	X	X	X
Government ownership		X		X		
Policy, programme development, and coordination (external)	X	X	X	X	X	X
Advocacy for partnerships					X	
Human resources (including training, capacity building and recruitment)	X			X	X	X
Internal planning, coordination and policy	X		X	X	X	X
Supply chain management	X	X	X	X	X	X
Supervision	X	X	X	X	X	
Monitoring and evaluation, and health information systems	X		X	X		X
Service delivery and referral system	X	X	X	X	X	X
Quality assurance for services	X	X	X	X	X	
Communication and social mobilisation	X	X	X	X	X	X
Monitoring and evaluation, and health information systems (pertaining to civil society capacity)	X		X			
Data quality		X		X		X
CHW* residency, training or transportation challenges		X				
Low utilization of iCCM* by communities		X				
Data management (including data use)				X	X	X
Health advocacy and resource mobilisation				X		
Advocacy for high-quality health services and data					X	
Incentives for CHWs					X	
Monitoring policy development (through TWG* or Task Force)					X	
Policy, advocacy and strategy at community levels						X
Human resources – engagement with community leaders						X

DRC – Democratic Republic of the Congo, CHW – community health workers, iCCM – integrated community case management, TWG – technical working group

### Synthesis of progress and recommendations

**Table 3** reports the vision statements and summarizes progress in RAcE project sites and recommendations for sustaining iCCM programmes. Each vision statement articulates a “big picture” goal for child health. The team in Malawi, which works with the most mature iCCM programme among RAcE project sites, outlined the most specific vision, calling out critical elements of iCCM programmes including personnel, supplies, and system supports.

**Table 3:** Vision statements, summary of progress, and recommendations

Democratic Republic of Congo	Malawi	Mozambique	Niger	Abia State, Nigeria	Niger State, Nigeria
**Vision statements**					
D'ici fin 2030, zéro décès lié au Paludisme, à la Diarrhée et à la Pneumonie des enfants de moins de cinq (5) ans grâce à la mise en place d'un système durable de PEC-C à tous les niveaux avec le concours de tous les partenaires impliqué*	By 2021 all children under five years of age in hard-to-reach areas with pneumonia, diarrhoea, and malaria receive prompt treatment around the clock from personnel who are trained, equipped, resourced, supervised, mentored, and practicing iCCM; residing in the catchment area with a good house, adequate drug supply, clinic structure, and functional referral system; using data for planning and decision making; within a knowledgeable and supportive community and enabling political environment to attain zero avoidable under-five deaths.	*Reduzida a mortalidade em crianças menores de cinco anos de idade, expandindo a cobertura de serviços de qualidade através de um sistema de saúde primário reforçado.†*	*D’ici 2026, un paquet complet de services curatifs, préventifs et promotionnels de qualité est rendu accessible à tous les enfants de moins de cinq (5) ans, d’une manière durable et équitable par des relais communautaires motivés dans toutes les communautés du Niger avec leur pleine participation.‡*	State government and stakeholders (community institutions, volunteers, local and international partners) will provide the resources (funds, environment, policy and capacity) to end preventable deaths of children 0-59 mo due to malaria, pneumonia and diarrhoeal diseases by 2030.	To implement iCCM in Niger State through institutionalizing sustainable support systems to reduce by 95% preventable deaths due to malaria, pneumonia, and diarrhoea in children between 0-59 mo, especially in hard to reach communities, by 2025.
**Progress as of May, 2017:**					
Each health zone integrated community health site coverage plans in operating plans.	HSA mapping activity conducted.	Not monitored due to project ending.	MSP continues to need support for transportation to supervise RCom in some districts.	The State Ministry of Health (SMOH) took over training on data management and use, and all refresher trainings for CORPs, community health extension workers (CHEWs), and local government area (LGA) focal persons.	LGA team members, the iCCM coordinator, and Malaria Consortium jointly conducted mentoring and coaching sessions for all CORPs and CHEWs.
All health zones had computers and tools to compile data.	Some facilities are using commodities intended for village clinics.		The national strategic plan for iCCM has not yet been adopted, delaying inclusion of iCCM costs in the state budget. [The plan was adopted in 2018.]	A formal data flow was established between the Abia State Primary Health Care Development Agency and state officials, and between state officials and the federal MOH.	SMOH was trained in data management.
Provincial MOH office took over monitoring and evaluation activities.	Discussions occurring to ensure that MOH procures all drugs.		Community leaders have verbally committed to supporting RComs, but there is no documentation about budgeting or other efforts.	Development of incentives programme and fundraising activities were planned.	All CORPs were supervised by CHEWs with standard supervision tools.
IRC still retaining ReCos and working with government partners to order, store, and distribute commodities and supplies.	Communication materials about iCCM were printed and planned for distribution at facilities.		Medicines are not consistently available at facilities.	Some Village Development Committees have not yet been established.	Uneven provision of incentives for CORPs by communities.
	Transportation for supervision is an ongoing challenge.		Referral system is not always accessible; slips are not consistently available at facilities.		Social mobilisation activities continued with support from MC.
	Refresher training for HSAs included how to complete referral and counter-referral forms.				RAcE project procured and distributed all commodities
	Lack of political will at district level.				
**Recommendations:**					
Identify people who would be responsible for strategic guidance and oversight of the iCCM programme, develop a harmonised plan and financing protocols for iCCM among donors, and identify and coordinate engagement with communities.	Engage communities through a consultative problem solving process.	Decentralize decision making to include contributions from civil society, community health committees, and other health system levels to improve demand for iCCM.	Find solutions to RCom remuneration and supervision. Explore cost sharing among key stakeholders.	Develop an incentives programme for CORPs.	Engage Ward Development Committees and Village Development Committees in commodity management to ensure that CORPs are fully stocked.
More thinking and planning is required regarding governance and financing issues for the health system overall, and for iCCM services within that system.	Avoid overburdening HSAs with other interventions that could fragment the iCCM programme.	Incorporate APEs formally in the MISAU human resource structure.	Adopt a validated national strategic plan for integrated community case management and child health.	Advocate with state officials to ensure the establishment of Village Development Committees, budgeting for iCCM programme costs, and supervision of community-based health workers.	Secure funding and commitment for social mobilisation activities.
Central MOH should provide more leadership.	Critically review the performance of current stock management programmes (c-stock).	Create a structure in MISAU to oversee iCCM activities, increase government ownership, and streamline technical support.	Identify and remedy bottlenecks in the supply chain.	Obtain lists of NGOs and other community-level actors to engage.	SMOH to take ownership of the HMIS.
Mobilizing funding for the recruitment of more ReCos.	Ensure IMCI Unit participates in development of community health strategy so that iCCM roadmap priorities are incorporated in it.	Improve collaboration in MISAU departments and across ministries to maximise efficiencies and leverage key resources for APEs and the iCCM programme.	Strengthen the referral system.	Define the roles and responsibilities of the members of the iCCM Task Force to aid in organising its efforts to work with the state government to sustain the iCCM programme.	Develop a human resource plan, including job descriptions for staff at all levels.
	Engage funding partners such as the Global Fund to assist with financing challenges.	Increase accountability to local communities to further enable MISAU and its partners to improve child health.	Formally situate the iCCM programme within the MSP so there is a clear line of support.	Establish an operations plan with a budget, a M&E plan, mentoring schedule for CORPs and CHEWs, state HMIS and procurement system for commodities.	Develop a data management plan.
	Implement supportive policies to address HSA residency issue.		Improve data collection and quality through standard protocols and tools and integrate data in HMIS.		Incorporate data use into M&E plan.
	Establish a leadership structure within MOH to support the iCCM programme.				Develop a community engagement strategy with social mobilisation and communication activities.
	Discuss HSA retention data at annual meetings and facilitate participant problem solving.				Develop a supply chain plan that addresses forecasting, procurement and distribution.
					Include iCCM as a core component in the State Primary Healthcare Strategy.
					Create terms of reference for iCCM Task Force.

CHW – community health worker, iCCM – integrated community case management, MOH – Ministry of Health, HSA – health surveillance assistant, CHEW – community health extension workers, MISAU - Ministerio da Saude, CORP - community-oriented resource person, SMOH – State Ministry of Health, APE – Agentes Polivalentes Elementares, NGO – non-governmental organization, HMIS – health management information system, RECO – *relais communautaires*

*Translation: By the end of 2030, zero deaths due to malaria, diarrhoea, and pneumonia of children under five (5) years through the establishment of a sustainable system of integrated community case management at all levels, with all involved partners.

†Translation: Reduced mortality among children under five years of age through expanded coverage of quality services in a strengthened primary health system.

‡Translation: By 2026, a comprehensive package of quality curative, preventative and promotional services is made available to all children under five (5) years of age, in a sustainable and equitable manner by community-based relays motivated in all communities of Niger with their full participation.

Community engagement, supply management, data processes, government leadership, and CHW remuneration or retention were recurrent themes in most project areas during the transition period and some examples are presented here. Social mobilisation activities were conducted with RAcE support in Niger State, but some Village Development Committees had yet to be established in Abia State and social mobilisation was carried out by a local NGO (Gracodev). In most areas, the RAcE project was still procuring and distributing supplies, although discussions with governments about taking over those tasks were under way. Some trainings in data collection and management had occurred, eg, in Niger State and in Malawi. In addition, health zones in DRC had tools to compile data. The Provincial MOH took over M&E activities in DRC and local government worked with the RAcE project to jointly mentor and coach CHWs in Niger State. However, a lack of district interest was noted in Malawi, and the national strategic plan for iCCM had not yet been adopted in Niger. Community support for CHWs was deemed important, but progress was uneven. In Niger State, some communities provided support through food and other incentives, while others did not. Community leadership did not move beyond verbal commitment in Niger. The RAcE project was still retaining ReCos in DRC. Although we could not monitor the transition in Mozambique due to the project ending, persistent threats to the APE programme in Mozambique have been noted, including donor-dependent funding for monthly incentives [13] and heavy workloads. In addition, stakeholders noted that MISAU has a limited capacity to manage, implement, and finance the iCCM programme.

Two recommendations for all project areas are to use the iCCM roadmap to guide future investments and efforts, and to update it regularly as the programmes mature. **Table 3** lists other recommendations addressing the common themes of community engagement, supply management, data processes, government leadership, and CHW remuneration or retention. Specifically, identifying champions and creating structures within the government to support iCCM programmes emerged as an important step in the immediate term to sustain the programmes. For example, given that iCCM was recently introduced in Abia State, focused advocacy efforts will be needed to ensure state ownership of the programme. Other strategies for engaging communities, improving data management, strengthening supply chains and supporting CHWs were also made.

## DISCUSSION

In this paper we reported the process and outputs of a sustainability planning initiative for RAcE project sites. We also compared roadmap priorities, progress against roadmaps, and recommendations among the project sites. We found that every project area identified priorities related to policy and coordination of external stakeholders including funders; supply chain management; service delivery and referral system; and communication and social mobilisation. Moreover, community engagement, supply management, data processes, government leadership, and CHW remuneration or retention were recurrent themes in most project areas during the transition period. Identifying champions and creating structures within the government to support iCCM programmes emerged as an important step in the immediate term to sustain the programmes.

Sustaining iCCM programmes can be crucial to sustaining improvements in child and community health outcomes in some settings, and is an emerging priority [8,11]. The framework we created incorporates the elements of a health system approach [41], while expanding service planning and delivery to include civil society partners and a broader consideration of the political and ecological environmental context. Identifying programme priorities and documenting them in a roadmap, along with deliberately planning for transition from an external implementer to a national system, may facilitate positive sustainability efforts and outcomes. These processes incorporate policy history and context, which have been deemed critical for national iCCM programme support [7], through the engagement of stakeholders at multiple levels and through multiple sectors. Although this approach should be formally tested, we have shown that adapting and using it in different contexts creates an inclusive and expansive (ie, multi-level and multisectoral) dialogue about systemic factors that influence the sustainability of a health service or programme.

The process of designing roadmaps included working with practical tools and guidance that facilitated thinking about specific issues related to implementing iCCM. This process included identifying critical challenges, involving multiple stakeholders, thinking across multiple sectors beyond the health system, establishing timeframes for achieving benchmarks, and building on established country or state strengths. Technical Working Groups (TWGs) formed at the conclusion of each workshop continued to advise roadmap updates in programme areas. Ideally, this group will be able to continually update the roadmap so that it is contextually current and responsive, a critical feature of working toward sustainability within a changing environment [38]. The feasibility of full transition from externally guided implementation to autonomous implementation in each project setting was variable, and in most countries it was evident that limited resources would not allow for the same level of iCCM services without donor funding. All RAcE projects were able to transition some roles, responsibilities, and activities for iCCM to the MOH and other local partners. Common aspects of programme implementation that were transitioned included monitoring and evaluation activities, supervision, training, and data management. It should be noted that, as LMIC economies grow, they may be able to assume a greater role in the administration of programmes currently funded by donors, although poorer countries are more susceptible to political corruption and violent conflict which undermine progress [42]. In addition, the fluctuations of global markets create uncertainty about sustained capacity to implement health and other programmes. Further, climate change has a disproportionate impact on LMIC [43].

Although the transition experienced some successes, a longer transition period would have been beneficial, meaning that planning for sustainability at the beginning of a project may have afforded a stronger “end game” for transition. Ideally, a sustainability framework should be used during programme planning to ensure that a programme is designed to be sustained [39]. This approach offers the advantage of providing an opportunity to build the capacity of national and local stakeholders in a measured and deliberate manner. In addition, convening key stakeholders to establish a TWG early in programme implementation would enable that group to exercise an important role in coordination and planning throughout programme implementation.

Finally, this process seeks to address practical realities to sustaining iCCM programmes through country-specific dialogue and solutions. While there is ongoing global dialogue about the best ways to finance iCCM programmes [44] and retain CHWs [45], countries and donors continue to grapple with how to ensure smooth transitions from external to internal funding and management. These discussions within countries naturally require multi-level and multisectoral conversations, which this sustainability planning process supports. But donors, too, could participate in sustainability planning dialogues and serve countries better by aligning investments with roadmap priorities, to ultimately move programmes closer to sustainment.

There are some limitations to this analysis. It was both comparative and summative; it was infeasible to analyze every roadmap element in detail. This approach necessarily tends toward superficiality; however, it is useful to look across programmes to review the emerging priorities for iCCM sustainability. Although limited conclusions can be drawn from the comparative analysis, and priorities and challenges are specific to context, the process and tools we described could be adapted for other settings, and should be formally tested. As iCCM grows as a health care delivery strategy in many countries, engaging stakeholders in processes to create TWGs and produce roadmaps could assist programme implementers with identifying and addressing the challenges that their programmes face, and ultimately sustaining health gains. In addition, our definition of sustainability is limited here to programme sustainability. The framework omits some relevant factors such as antimicrobial resistance and does not specify elements such as climate change, which is becoming increasingly important for health service planning. Further, future studies may consider power dynamics among stakeholders to elucidate relevant factors affecting sustainability [46,47], and macro-level factors that can counteract system strengthening efforts, such as competing priorities and the hierarchical structure of personnel roles in a system [48]. Global reviews of iCCM implementation will continue to inform sustainability planning by identifying emerging factors to incorporate in relevant frameworks [49].

## CONCLUSIONS

In conclusion, the expectation for the sustainability of a health service is that the local system that produces health (inclusive of policy makers, programme implementers and service providers, and community members) is robust and resilient enough to maintain health coverage and outcome gains while adapting to changing conditions. Embarking on a process to plan for the sustainability of iCCM services optimizes investments in the programme by ensuring that life-saving curative services will continue to be available to children in hard-to-reach areas when funding and other conditions change. Areas where work remains to increase the likelihood of iCCM programme sustainability included political leadership; supply chain management; human resource capacity, supervision and retention; data management; and, community engagement. Future investments in iCCM programmes should assist country teams to address these issues.

## Additional material

Online Supplementary Document

## References

[1] Rabin BA Brownson RC Haire-Joshu D Kreuter MW Weaver NL A glossary for dissemination and implementation research in health. J Public Health Manag Pract. 2008;14:117-23. 10.1097/01.PHH.0000311888.06252.bb 18287916

[2] United States Agency for International Development and Maternal and Child Health Integrated Program. Integrated community case management of childhood illnesses: documentation of best practices and bottlenecks to program implementation in the Democratic Republic of Congo (DRC). Available: https://www.mchip.net/sites/default/files/mchipfiles/DRCLongEnglish.pdf. Accessed: 20 May 2018.

[3] Sarriot EG Swedberg E Ricca J Pro-sustainability choices and child deaths averted: from project experience to investment strategy. Health Policy Plan. 2011;26:187-98. 10.1093/heapol/czq042 20823216

[4] Goldberg J Country ownership and capacity building: the next buzzwords in health systems strengthening or a truly new approach to development? BMC Public Health. 2012;12:531. 10.1186/1471-2458-12-531 22818046 PMC3461461

[5] Bao J Rodriguez DC Paina L Ozawa S Bennett S Monitoring and evaluating the transition of large-scale programs in global health. Glob Health Sci Pract. 2015;3:591-605. 10.9745/GHSP-D-15-00221 26681706 PMC4682584

[6] World Health Organization, United Nations Children’s Fund. Joint statement on integrated community case management: An equity-focused strategy to improve access to treatment services for children. Available: https://www.unicef.org/health/files/iCCM_Joint_Statement_2012.pdf. Accessed: 20 May 2018. 10.4269/ajtmh.2012.12-0221 PMC3748523 23136272

[7] George A Young M Nefdt R Basu R Sylla M Clarysse G Community health workers providing government community case management for child survival in sub-Saharan Africa: who are they and what are they expected to do? Am J Trop Med Hyg. 2012;87:85-91. 10.4269/ajtmh.2012.11-0757 23136282 PMC3748527

[8] Rasanathan K Muniz M Bakshi S Kumar M Solano A Kariuki W Community case management of childhood illness in Sub-Saharan Africa: findings from a cross-sectional survey on policy and implementation. J Glob Health. 2014;4:020401. 25520791 10.7189/jogh.04.020401 PMC4267096

[9] Young M Wolfheim C Marsh DR Hammamy D World Health Organization/United Nations Children’s Fund joint statement on integrated community case management: An equity-focused strategy to improve access to essential treatment services for children. Am J Trop Med Hyg. 2012;87:6-10. 10.4269/ajtmh.2012.12-0221 23136272 PMC3748523

[10] Boschi-Pinto C Labadie G Dilip TR Oliphant N Dalglish SL Aboubaker S Global implementation survey of Integrated Management of Childhood Illness (IMCI): 20 years on. BMJ Open. 2018;8:e019079. 10.1136/bmjopen-2017-019079 30061428 PMC6067364

[11] Daelmans B Seck A Nsona H Wilson S Young M Integrated community case management of childhood illness: What have we learned? Am J Trop Med Hyg. 2016;94:571-3. 10.4269/ajtmh.94-3intro2 26936992 PMC4775893

[12] Bennett S George A Rodriguez D Shearer J Diallo B Konate M Policy challenges facing integrated community case management in Sub-Saharan Africa. Trop Med Int Health. 2014;19:872-82. 10.1111/tmi.12319 24750516 PMC4282431

[13] Chilundo BG Cliff J Mariano A Rodriguez D George A Relaunch of the official community health work program in Mozambique: is there a sustainable basis for iCCM policy? Health Policy Plan. 2015;30:ii54-64. 10.1093/heapol/czv014 26516151 PMC4625760

[14] Daviaud E Besada D Leon N Rohde S Sanders D Oliphant N Costs of implementing integrated community case management (iCCM) in six African countries: Implications for sustainability. J Glob Health. 2017;7:010403. 10.7189/jogh.07.010403 28702174 PMC5502705

[15] George A Rodriquez DC Rasanathan K Brandes N Bennett S iCCM policy analysis: Strategic contributions to understanding its character, design and scale up in sub-Saharan Africa. Health Policy Plan. 2015;30:ii3-11. 10.1093/heapol/czv096 26516148

[16] Hamer DH Marsh DR Peterson S Pagnoni F Integrated community case management: next steps in addressing the implementation research agenda. Am J Trop Med Hyg. 2012;87:151-3. 10.4269/ajtmh.2012.12-0505 23136291 PMC3748516

[17] Vogus A Graff K PEPFAR transitions to country ownership: review of past donor transitions and applications of lessons learned to the eastern Caribbean. Glob Health Sci Pract. 2015;3:274-86. 10.9745/GHSP-D-14-00227 26085023 PMC4476864

[18] Oberth G Whiteside A What does sustainability mean in the HIV and AIDS response? Afr J AIDS Res. 2016;15:35-43. 10.2989/16085906.2016.1138976 26785676

[19] Dalglish SL George A Shearer JC Bennett S Epistemic communities in global health and the development of child survival policy: a case study of iCCM. Health Policy Plan. 2015;30:ii12-25. 10.1093/heapol/czv043 26516146

[20] Rodríguez DC Shearer J Mariano ARE Juma PA Dalglish SL Bennett S Evidence-informed policymaking in practice: country-level examples of use of evidence for iCCM policy. Health Policy Plan. 2015;30:ii36-45. 10.1093/heapol/czv033 26516149 PMC4625759

[21] Dalglish SL Rodríguez DC Harouna A Surkan PJ Knowledge and power in policy-making for child survival in Niger. Soc Sci Med. 2017;177:150-7. 10.1016/j.socscimed.2017.01.056 28167340

[22] Sarriot E Morrow M Langston A Weiss J Landegger J Tsuma L A causal loop analysis of the sustainability of integrated community case management in Rwanda. Soc Sci Med. 2015;131:147-55. 10.1016/j.socscimed.2015.03.014 25779620

[23] Strachan C Wharton-Smith A Sinyangwe C Mubiru D Ssekitooleko J Meier J Integrated community case management of malaria, pneumonia and diarrhoea across three African countries: a qualitative study exploring lessons learnt and implications for further scale up. J Glob Health. 2014;4:020404. 10.7189/jogh.04.020404 25520794 PMC4267083

[24] Martin NA Hulland KRS Dreibelbis R Sultana F Winch PJ Sustained adoption of water, sanitation and hygiene interventions: systematic review. Trop Med Int Health. 2018;23:122-35. 10.1111/tmi.13011 29160921

[25] Ministère du Plan et Suivi de la Mise en oeuvre de la Révolution de la Modernité (MPSMRM), Ministère de la Santé Publique (MSP), ICF International. Chapter 10: child health. Democratic Republic of Congo demographic and health survey 2013-14: key findings. Rockville, MD, USA: MPSMRM, MSP, and ICF International; 2014:144–156.

[26] United States Agency for International Development and Maternal and Child Health Integrated Program. Integrated community case management of childhood illnesses: documentation of best practices and bottlenecks to program implementation in the Democratic Republic of Congo (DRC). Available: https://www.mchip.net/sites/default/files/mchipfiles/DRCLongEnglish.pdf. Accessed: 20 May 2018.

[27] World Health Organization. Democratic Republic of Congo: WHO statistical profile. Available: http://www.who.int/gho/countries/cod.pdf?ua=1. Accessed: 20 May 2018.

[28] National Statistical Office/Malawi and ICF. Malawi demographic and health survey 2015-16. Zomba, Malawi: National Statistical Office and ICF; 2017.

[29] World Health Organization Regional Office for Africa, Global Health Observatory. Malawi: WHO statistical profile. Available: http://www.who.int/gho/countries/mwi.pdf?ua=1. Accessed: 20 May 2018.

[30] Nsona H Mtimuni A Daelmans B Callaghan-Koru JA Gilroy K Mgalula L Scaling up integrated community case management of childhood illness: update from Malawi. Am J Trop Med Hyg. 2012;87:54-60. 10.4269/ajtmh.2012.11-0759 23136278 PMC3748522

[31] Countdown to 2015: Maternal, Newborn, and Child Survival, 2015.

[32] Ministerio da Saude. Relatorio Anual das Actividades do Programa de Agentes Polivalentes Elementares (APEs) do Ano 2013. Maputo, Mocambique: Ministry of Health; 2014. [Translation: 2013 Annual Report of the APE Programme].

[33] da Saude M. (MISAU)/Moçambique, Instituto Nacional de Estatística (INE)/Moçambique, ICF International. Moçambique Inquérito Demográfico e de Saúde 2011. Calverton, MD, USA: MISAU/Moçambique, INE/Moçambique, and ICF International; 2013.

[34] United Nations Development Programme. Data on under-five mortality rate (per 1,000 births). Available: http://hdr.undp.org/en/indicators/57506. Accessed: 20 May 2018.

[35] United Nations Children’s Fund Data and Analytics Section, Division of Data, Research and Policy. Child mortality estimates: global and regional child deaths by cause. Available: https://data.unicef.org/topic/child-survival/under-five-mortality. Accessed: 20 May 2018.

[36] Institut National de la Statistique (INS)/Niger, ICF International. Niger Enquête Démographique et de Santé et à Indicateurs Multiples (EDSN-MICS IV) 2012. Calverton, Maryland, USA: INS/Niger and ICF International; 2013.

[37] National Population Commission (NPC) [Nigeria], ICF International. Nigeria demographic and health survey 2013. Abuja, Nigeria, and Rockville, MD, USA: NPC and ICF International; 2014.

[38] Chambers DA Glasgow RE Stange KC The dynamic sustainability framework: addressing the paradox of sustainment amid ongoing change. Implement Sci. 2013;8:117. 10.1186/1748-5908-8-117 24088228 PMC3852739

[39] Sarriot E, Ricca J, Yourkavitch J, Ryan L, et al. Taking the long view: A practical guide to sustainability planning and measurement in community-oriented health programming et al. Taking the long view: A practical guide to sustainability planning and measurement in community-oriented health programming. Calverton, MD: Macro International Inc.; 2008.

[40] Sarriot E Kouletio M Community health systems as complex adaptive systems: ontology and praxis lessons from an urban health experience with demonstrated sustainability. Syst Pract Action Res. 2015;28:255-72. 10.1007/s11213-014-9329-9

[41] McGorman L Marsh DR Guenther T Gilroy K Barat LM Hammamy D A health systems approach to integrated community case management of childhood illness: methods and tools. Am J Trop Med Hyg. 2012;87:69-76. 10.4269/ajtmh.2012.11-0758 23136280 PMC3748525

[42] Radelet S. The great surge: the ascent of the developing world. New York, NY: Simon and Schuster; 2015.

[43] World Health Organization. Climate change and health fact sheet. Available: http://www.who.int/en/news-room/fact-sheets/detail/climate-change-and-health. Accessed: 30 September 2018.

[44] Management Sciences for Health. iCCM costing and financing tool: implementation manual and user guide. 2010. Available: https://www.msh.org/sites/msh.org/files/iccm_costing_and_financing_tool_userguide_version1.0.pdf. Accessed: 29 July 2018.

[45] World Health Organization. Increasing access to health workers in remote and rural areas through improved retention: global policy recommendations. Available: https://ccmcentral.com/wp-content/uploads/2014/04/Improving-CHW-Retention-Policy-recommendations_WHO_2010.pdf. Accessed: 29 July 2018. 23741785

[46] Dalglish SL Surkan PJ Diarra A Harouna A Bennett S Power and pro-poor policies: the case of iCCM in Niger. Health Policy Plan. 2015;30 Suppl 2:ii84-94. 10.1093/heapol/czv064 26516154

[47] Dalglish SL Methods for the strategic review of programmes for integrated management of childhood illness and community cases. BMJ. 2018;362:k2989. 10.1136/bmj.k2989 30061099 PMC6063343

[48] Thomas JC Contextual factors affecting health information system strengthening. Glob Public Health. 2017;12:1568-78. 10.1080/17441692.2016.1256419 27841079

[49] Dalglish SL Sriram V Scott K Rodríguez DC A framework for medical power in two case studies of health policymaking in India and Niger. Glob Public Health. 2019;14:542-54. 10.1080/17441692.2018.1457705 29616876

