# Supplementary material for: Integrated community case management: planning for sustainability in five African countries
Source: J Glob Health. 2019 Jun 25;9(1):010802. doi: 10.7189/jogh.09.010802 (PMC6596361; doi:10.7189/jogh.09.010802)
Supplement: Online Supplementary Document [file jogh-09-010802-s001.pdf]

J Glob Health 2019; 9: 010802

Supplemental File S1. Sustainability Framework for iCCM in Malawi

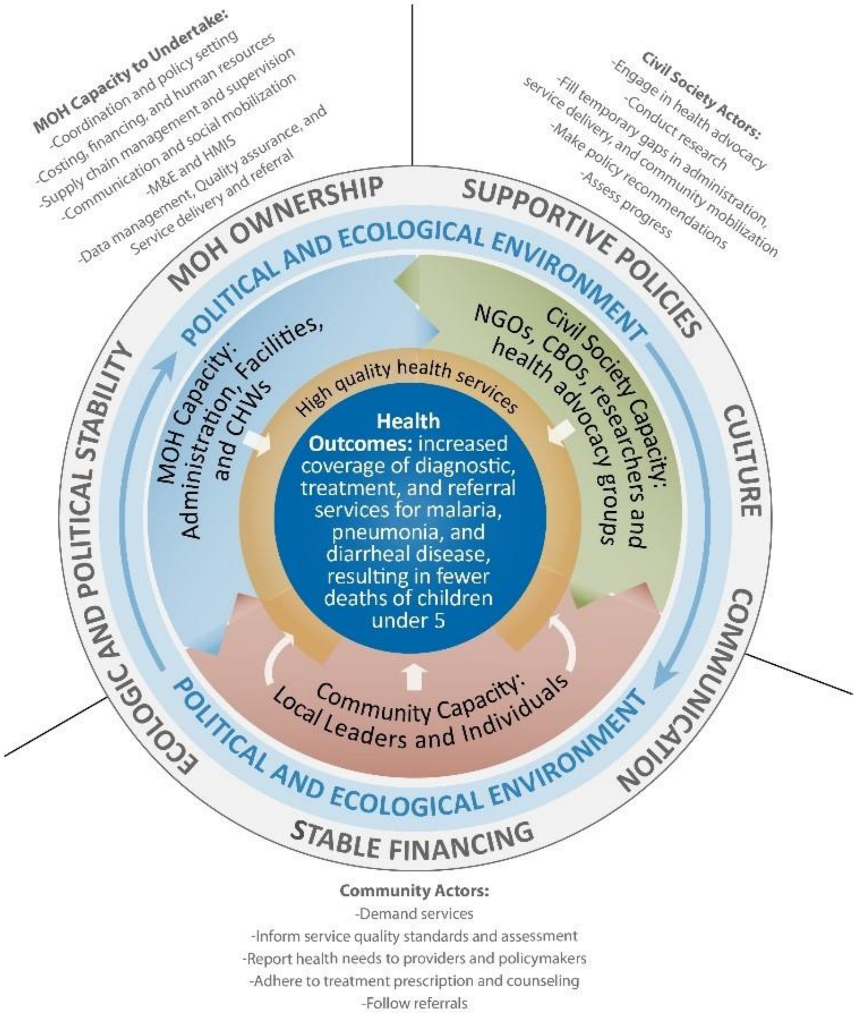

Supplemental File S.2. Workshop Participants

**Participants**	**Democratic Republic of Congo***	**Malawi**	**Mozambique**	**Niger**	**Abia State, Nigeria**	**Niger State, Nigeria**
Ministry of Health (central, or state for Nigeria)	9	7	10	11	7	21
Ministry of Health (provincial or local)	2	45	12	41	7	21
NGO staff (grantee)	5	9	13	6	10	8
Other Ministry staff (central, or state for Nigeria)	10	0	2	0	8	2
Other Ministry staff (provincial or local)	2	0	0	0	0	5
ICF (facilitation)	3	3	2	3	3	3
WHO	3	3	2	3	2	5
Other experts	3	0	0	0	1	9
Other NGOs	0	8	1	2	3	7
Community representatives	0	0	0	0	15	0
Other donors	1	4	1	2	0	1
**Total**	**38**	**79**	**43**	**68**	**55**	**82**

*This workshop occurred during a time of civil unrest which prevented some stakeholders from participating.

Supplemental File S3. Sample Workshop Agenda

National Sustainability Planning Workshop for Integrated Community Case Management and RAcE Transition—*Draft Agenda*

Goal: To establish integrated community case management (iCCM) as a core component of Ministry of Health (MOH) service delivery

Key Objectives:

1. To draft a roadmap for institutionalizing iCCM
2. To create a draft transition plan in support of the roadmap for transitioning Rapid Access Expansion (RAcE) iCCM programme to MOH

Requested/anticipated participants:

- National and local MOH
- WHO country team
- Grantee country team
- iCCM technical working group members
- Other donors
- Any other stakeholders (determined at Abuja meeting)

Description:

- Participatory workshop, lasting three days

Workshop Day 1: 8:30 a.m.–5:30 p.m.

Time	Agenda	Presenters
**Morning**	• Introduction (chair) (15 min)<br>• Welcome address (15 min)<br>• Objectives of the meeting (30 min)	World Health Organization (WHO)<br>MOH<br>WHO
	**Objective 1: To draft a roadmap for institutionalising iCCM**	
	• iCCM overview, national vision, and progress (1 hour)<br>***Tea break*** (30 min)<br>• Presentation and discussion of the RAcE programme results (1 hour)<br>• Review of workshop agenda and introduction of visioning activity (20 min)	MOH<br><br>Grantee<br><br>ICF International
	Lunch 1 p.m.	
**Afternoon**	• Visioning activity (large group to start; break into small groups that present back to large group) (1 hour)<br>***Tea break*** (30 min)	ICF International

Time	Agenda	Presenters
	• Orientation to sustainability theory, RAcE sustainability framework, activities, and timelines (2 hours)<br>• Summary of Day 1 (15 min)	ICF International<br><br>Rapporteur

Workshop Day 2: 8:30 a.m.–5:30 p.m.

Time	Agenda	Presenters
	**Objective 1: To draft a sustainability roadmap for institutionalizing iCCM (cont'd)**	
**Morning**	• Recap of Day 1 with questions/discussion (30 min)<br>• Transition readiness discussion and introduction to roadmap followed by small group activity to discuss health system components and transition readiness benchmarks for iCCM (2 hours)<br>***Tea break*** (30 min)<br>• Small group work to develop roadmap and milestones (1 hour)	WHO<br>ICF International Consultant<br><br>ICF International Consultant
	Lunch 1 p.m.	
**Afternoon**	• Small group work to develop roadmap and milestones (cont'd) (1.5 hours)<br>***Tea break*** (30 min)<br>• Small group work to develop roadmap and milestones (cont'd) (1 hour)<br>• Summary of Day 2 (15 min)	ICF International Consultant<br><br>Rapporteur

Workshop Day 3: 8:30 a.m.–5:30 p.m.

Time	Agenda	Presenters
**Morning**	• Large group harmonization of roadmap (2 hours)<br>***Tea break*** (30 min)<br>• Agree on next steps to finalise the roadmap (30 min)<br>• Review of progress and introduction to transition plan template (30 min)	ICF International Consultant<br><br>MOH<br>ICF International Team
	Lunch 12:30 p.m.	
	**Objective 2: To create a draft transition plan in support of the sustainability roadmap for transitioning WHO- and nongovernmental organisation-led support for the iCCM programme to MOH**	
**Afternoon**	• Instructions for completing the transition plan template (15min)	ICF International Team and Grantee

Time	Agenda	Presenters
	• Group work to develop transition plan (1.5 hours)<br>***Tea break*** (30 min)<br>• Presentation on draft transition plans (1 hour)<br>• Agree on next steps to finalise the transition plan (30 min)<br>• Summarise progress and clarify next steps (15 min)	ICF Consultant and Grantee<br>MOH

Supplemental File S4. Case study with monitoring information for Niger State, Nigeria

Background

In Niger State, and throughout Nigeria, the health sector operates at three tiers—primary, secondary, and tertiary—with primary health care services provided as mainly preventive, promotive, protective, restorative, and rehabilitative; secondary health care services as mainly curative and, to some degree, preventative, protective, and rehabilitative; and tertiary health care services as specialised, curative, and restorative (Niger State Ministry of Health 2009). Health care services in Niger State have recently received more funding support, which has enabled the state to construct additional facilities and renovate existing ones (Niger State Ministry of Health 2009). The primary challenge that the state health system faces is inadequate human resources, both in terms of quantity and quality (Niger State Ministry of Health 2009).

The RAcE programme was launched on November 1, 2013, in Niger State, with the aim of delivering iCCM services in hard-to-reach communities and building State MOH and Primary Health Care Development Agency (PHCDA) capacity to introduce and implement community-based services. As a first step, the Federal Ministry of Health established the National iCCM Task Force and sub-committees, followed by the development of national guidelines on iCCM and updated relevant policies and strategies to incorporate iCCM in national policy. The sustainability planning component of the programme was implemented to maintain investments in iCCM in the state, given that the RAcE programme pioneered iCCM implementation in Nigeria and led to the adoption of national guidelines for iCCM implementation.

Identification of a Local Consultant

To begin, ICF identified a consultant to be the key point of contact between the ICF sustainability team, the Niger SMOH, and the implementing partner. It was important that the consultant be independent (unaffiliated with the MOH or the implementing partner) because he or she would facilitate discussions between the TWG and stakeholders. The consultant's scope of work included participating in a sustainability concepts training facilitated by ICF, organising the country workshop, gathering inputs from key stakeholders to finalise the roadmap, monitoring progress against the roadmap, contributing to the final sustainability synthesis report, and presenting the findings from that analysis to stakeholders. ICF reviewed candidates' resumes collected through referrals to identify a consultant who had suitable past experience working with key stakeholders and was able to reliably manage and coordinate logistics in the country.

Sustainability Workshop: Planning

Planning began more than three months before the workshop and was initiated at the project inception meeting in Abuja, Nigeria, during which stakeholders from Niger State conducted a mapping exercise with the SMOH. Following the inception meeting, ICF's local consultant, WHO, SMOH, and implementing partners met several times to finalise the workshop agenda and confirm logistics for both Abuja and Niger State. WHO representatives in Niger State formally invited workshop participants.

ICF trained the local consultant, drafted the workshop agenda, and created facilitators' guidance and other workshop materials, such as presentations, group activity

instructions, and templates for the roadmaps and transition plans. Lessons learned from sustainability workshops held in other countries implementing the RAcE programme also informed the development of workshop materials.

Planning meetings in Niger State included representatives from the grantee (Malaria Consortium), WHO, SMOH, and ICF to discuss the format of the workshop, finalise the agenda, and identify relevant focal points and *rapporteurs* to take notes during the workshop. A core group of participants and facilitators also met at the end of each workshop day to debrief and agree on agenda adjustments for the following day.

Sustainability Workshop: Accomplishments

The workshop was held from October 4 to 6, 2016. Stakeholders attending the workshop created a vision of iCCM sustainability for Niger State; drafted a roadmap through 2025 to attain that vision; and drafted a transition plan to guide the transition of the RAcE iCCM programme to the SMOH for the last year of the RAcE programme, which was, effectively, the first year of the roadmap. The transition plan outlined key RAcE activities for the Federal MOH and SMOH to take over by January 2018. These transition activities were accomplished:

- Developed an iCCM annual operational plan for the state to be implemented in the 25 Local Government Areas (LGA) of the state.
- Developed an iCCM annual operational plan for the six LGAs that implemented the RAcE project.
- Ensured that a budget line was created for iCCM implementation in the 2018 state budget.

- Aired media jingles through outlets in the state.
- Distributed iCCM medicines and other consumables to the reference health facilities and to the CORPs.
- Trained six LGA iCCM focal persons and monitoring and evaluation officers to generate utilization data with a spreadsheet.

The first morning focused on the formal opening of the workshop with a presentation of the workshop objectives, an overview of the iCCM programme, and a discussion of the RAcE programme. The Honourable Commissioner for Health presented the keynote address. In the afternoon session, ICF’s Sustainability Team provided an explanation of systems dynamics and complex systems analysis, gave a brief overview of the components of the sustainability framework adapted for RAcE and its link to various aspects of iCCM programme implementation, and introduced the group visioning activity. The purpose of visioning was to build consensus for a common vision of a sustainable iCCM programme through small groups that would coalesce in a shared vision in the large group.

The second day began with a recapitulation of discussions from the first day and a review of the vision statements developed by each small group. All groups then finalised their vision statements in text and drawings, and those working documents were hung on the wall for a “gallery walk” exercise to synthesise contributions in a single vision statement. This activity allowed participants to see the work of all the groups and was followed by a plenary discussion. The groups presented their vision statements and identified actors and actions required to actualise them (Fig S4).

Fig S4. Visioning work

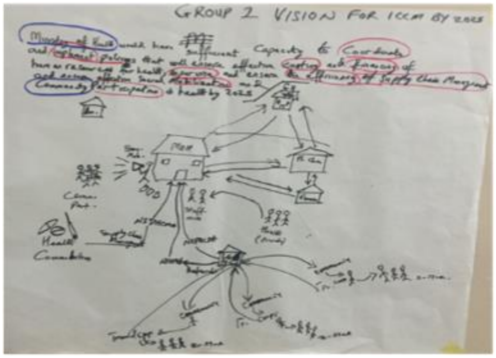

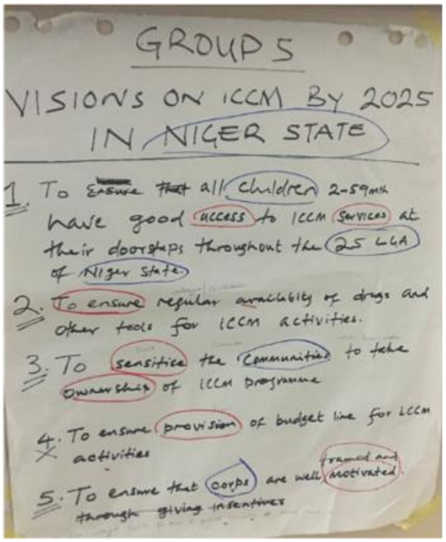

ICF facilitated consensus around a single vision statement for iCCM in Niger State:

To implement iCCM in Niger State through institutionalizing sustainable support systems to reduce by 95% preventable deaths due to malaria, pneumonia, and diarrhoea in children between 0-59 months, especially in hard to reach communities, by 2025.

During the afternoon session of the second day, the groups began roadmap development to achieve the vision, organising activities according to the components of the sustainability framework. The SMOH, WHO, and Malaria Consortium determined group composition based on participants’ previous iCCM and RAcE programme work experience and their job roles and responsibilities. Each group was assigned a component from the sustainability framework (high-quality health services and data, MOH capacity, community capacity, civil society capacity, or political and ecological environment), and the groups were tasked with discussing issues and possible solutions for the sustainability of the assigned component, as relevant to iCCM implementation in Niger State. At the end of the second day, two groups presented their roadmap components to participants.

The other groups presented their roadmap components at the beginning of the third day. In the second half of the third day, WHO presented the results from the RAcE surveys that highlighted key trends in iCCM programme implementation for Niger State. Following this, groups outlined a transition plan that would address challenges and sustain achievements, and then developed and presented lists of critical needs for a successful transition.

Next Steps

During the workshop, the iCCM committee chairperson identified participants who should be part of the state's TWG to oversee and continue the roadmap development process. After the workshop, the core planning group (ICF, WHO, and Malaria Consortium) and the newly identified TWG participants met to identify next steps for completing the roadmap and transition plan.

Post-workshop Roadmap Finalisation Process

After the workshop, ICF assembled the outputs and consolidated inputs to form the first draft of the roadmap. The roadmap then went through several revisions, with ICF providing suggestions to make activities more specific and actionable and the TWG coordinating responses. The TWG also gathered inputs on timelines and details of activities from relevant stakeholders representing the SMOH, the State PHCDA, and LGAs who would be responsible for specific activities as part of their efforts to finalise the roadmap. ICF's consultant participated in the TWG meetings and ensured that the revisions and feedback were completed in a timely manner.

The roadmap contained the following sections:

- Background on infant and child health, iCCM, and related policies in Nigeria
- Introduction to the RAcE project
- Purpose of the iCCM roadmap
- Summary of the roadmap development process
- A table listing priorities organized by components of the sustainability framework, persons or organisations responsible for leading the activities, and a general timeframe for completing the activities

Core components of the iCCM roadmap were determined through multiple consultations with key stakeholders at national, district, and community levels as described above. The core components of the iCCM roadmap are as follows:

- **Priority** issues determined by stakeholders to be essential to successful iCCM delivery
- **Activities** to address priority issues
- **Actors responsible** to lead and contribute to these activities
- **Milestones** for assessing progress towards addressing the issues

Post-workshop Transition Plan Development Process

The transition plan was designed to be a detailed workplan for roadmap activities due to be completed before the end of the RAcE project. Although the roadmap was developed as a SMOH document, the transition plan was finalised by Malaria Consortium, and transition efforts were monitored by ICF's consultant. Malaria Consortium finalised the transition plan after ICF combined the inputs of the workshop participants. The plan mirrored the roadmap in that there were actions for each

component, with responsible actors and milestones identified. ICF evaluated the quality of the transition plan to determine whether:

- The format of the plan was appropriate and could be used for monitoring purposes.
- Content was complete and activities were described in adequate detail with appropriate SMOH participation.
- Timelines were appropriate and indicated approximately when transition activities would occur according to the incremental benchmarks in the total period of performance.

ICF monitored the transition activities monthly from February through April 2017 with a customized monitoring tool derived from the transition plan. ICF met with Malaria Consortium and used the tool to record the progress of the activities in the plan. Supplementary File S.4 is a table of information based on the last monitoring update in April 2017. The monitoring process fostered accountability on the part of the grantee regarding its transition plan activities.

Dissemination and Next Steps

A two-day meeting was held in Minna, Niger State on August 1 and 2, 2017, with the goals of disseminating the WHO RAcE project evaluation survey results and transition monitoring information, and updating the sustainability roadmap with stakeholders. The meeting was attended by 80 stakeholders from WHO, the Federal Ministry of Health, the National PHCDA, the National Malaria Elimination Programme,

the Niger SMOH, the Niger State PHCDA, implementing LGAs (chairs and staff), Malaria Consortium, and other NGOs and faith-based organisations.

Group work sessions provided an opportunity to discuss the results presented and update the roadmap accordingly. Revisions included the addition of new activities and further elaborating on existing activities. After the meeting, the TWG compiled the edits. The final roadmap was signed by the Niger State Honourable Commissioner of Health in July 2017. Because the roadmap is considered a living document, however, further updates were made during the RAcE dissemination meeting, which was held in Abuja in October 2017.

Synthesis of Progress

The synthesis report identified 16 elements from the roadmap: policy related to advocacy and strategy; policy related to finance and budget; policy related to coordination and planning; coordination and planning for the iCCM Task Force; human resources supervision and training; communication and social mobilisation; mentoring and coaching; service delivery; data quality; data management, use, and availability; data management monitoring and evaluation tools; supply chain management and logistics; policy advocacy and strategy at the community level; monitoring and evaluation and surveys; community engagement; and human resources—engagement with community leaders. LGA team members, the iCCM coordinator, and Malaria Consortium jointly conducted mentoring and coaching sessions for all CORPs and CHEWs during the transition period. The SMOH acquired training in data management (Table S4).

Priorities for the near term include engaging Ward Development Committees and VDCs in commodity management to ensure that CORPs are fully stocked; securing funding and commitment for social mobilisation activities; SMOH taking ownership of the HMIS; and developing a human resource plan, including job descriptions for staff at all levels. As a new iCCM programme, this one is particularly vulnerable to faltering due to weaknesses in the health system. Although state commitment to the programme is strong, additional resources are needed to further systematise elements of the iCCM programme and ensure that it is integrated with the larger health system. Most of the activities contained in the roadmap are currently being implemented by state actors.

Table S4. Monitoring information

Action	Transition monitoring update, 28 April 2017
Conduct competency assessment for CORPs	Training commenced by MC and SMoH in April but was halted because WHO suggested a review of the tools. Review is ongoing. MC provided financial and technical support.
Quarterly mentoring and coaching of LGA team, supervisors, CORPs Head and CORPs	RAcE team, State iCCM team, IMCI Coordinator, LGA team, supervisors (and CORPs Head) to conduct visits. One visit was conducted, resulting in 85% of all CORPs and CORPs supervisors mentored and coached. Key skills targeted: case management, documentation, supervision and logistics management. MC provided financial and technical support.
Support CORPs supervision across the project LGAs	MC and LGA supervisors (CHEWs) supervised 100% of CORPs (n=1320) in the first quarter of the 2017.
Data management – Train iCCM State & LGA implementation team on data management	The state and LGA implementation team was trained. MC provided financial and technical support for the training. MC will strengthen MOH capacity to conduct

Action	Transition monitoring update, 28 April 2017
	continuous education and capacity building on data management.
Production and use of ICCM M&E tools(soft and hard copies) such as CORPs daily register, facility summary register, sick child recording forms, supervisory checklist, referral note to improve quality of care.	Data tools produced and used to improve quality of care (CORPs daily register, facility summary register, sick child recording forms, supervisory checklist, referral note). MC provided financial and technical support.
Operationalization of the Community DHIS	Data to be uploaded in the national HMIS. This is coordinated at the national level and is in progress.
Support Quarterly State iCCM taskforce meeting to provide strategic guidance and oversight in all matters concerning iCCM. This includes building synergy through networking at the meetings, joint planning and funding, increasing access and maximize resources, driving the process of initiating and institutionalizing iCCM in Niger State and ensuring the implementation of decisions taken	Quarterly task force meetings held (1 quarterly meeting reports submitted). MC hosted the meetings but will transition this to MOH.
Support Quarterly meeting of WDCs/ VDCs (community) to drive the ownership of iCCM programme	Report of meetings documented, follow-up actions and recommendations implemented by responsible persons /institutions to improve programme implementation: 70% completed. SPHCDA will organize future meetings and implement recommendations.
Support the review and development Annual Operational planning (AOP)	AOP 2017/2018 processes are in place. Review proposed for August, 2017. MC to participate in the meeting and provide financial and technical support to the AOP review.
Support the training of resource mobilization(including costing of iCCM Plans)	Processes in place; training proposed for June, 2017. MC to provide financial support for the training.
Support the conduct of State, LGA and community level resource mobilization activities to fund 15% of the State iCCM plan	Resource Mobilization activities conducted. 15% resources need to fund ICCM mobilized. Currently there is some level of ongoing community resource mobilization. MC will provide technical support to train state and LGA officials on

Action	Transition monitoring update, 28 April 2017
	resource mobilization. MC will partner with state and LGAs to institutionalize the iCCM programme in their activities.
Support the conduct of social mobilization activities in iCCM communities across the State (State, LGAs and community levels) to increase demand for services, facilitate community involvement and ownership	Estimated 26% State, LGAs and community levels social mobilization activities completed. MC provides stipends and technical guidance to the social mobilisers who carry out these activities.
Support the airing of 20 Radio jingles daily and two magazine programmes monthly	20 Radio jingles aired daily; two radio magazine programmes aired monthly to increase awareness, demand for services and education on ICCM. MC engaging MOH to transition this activity.
Support the ACSM committee to monitor the use of social and behavior change communication materials towards increasing demand for services, provision of information and increasing knowledge of ICCM at State, LGA and Community levels.	The initial budget earmarked for this activity was low but this has been reviewed. A consultant will be hired to work on the SBCC materials. MC works in partnership with SMoH on the design and production of the materials.
Procure, store and distribute iCCM Commodities to health facilities and end users using existing system	iCCM commodities procured, stored and distributed to end users using existing systems. MC procures and distributes needed commodities to 57 Reference Health Facilities across the 6 project LGAs in the State. MC will support SMoH to quantify the needed commodities and consumables needed for the implementation.
Support the conduct of community dialogue and mobilization at community level.	Estimated 27% of communities are fully able to access iCCM services and drugs. MC supports these activities by providing stipends and technical support to the social mobilisers.
